# Predicting *Mycobacterium tuberculosis* Complex Clades Using Knowledge-Based Bayesian Networks

**DOI:** 10.1155/2014/398484

**Published:** 2014-04-23

**Authors:** Minoo Aminian, David Couvin, Amina Shabbeer, Kane Hadley, Scott Vandenberg, Nalin Rastogi, Kristin P. Bennett

**Affiliations:** ^1^Department of Computer Science, Rensselaer Polytechnic Institute, Troy, NY 12180, USA; ^2^WHO Supranational TB Reference Laboratory, Tuberculosis and Mycobacteria Unit, Institut Pasteur de la Guadeloupe, 97183 Abymes, Guadeloupe, France; ^3^Department of Computer Science, Siena College, Loudonville, NY 12180, USA; ^4^Department of Mathematical Sciences, Rensselaer Polytechnic Institute, Troy, NY 12180, USA

## Abstract

We develop a novel approach for incorporating expert rules into Bayesian networks for classification of *Mycobacterium tuberculosis* complex (MTBC) clades. The proposed knowledge-based Bayesian network (KBBN) treats sets of expert rules as prior distributions on the classes. Unlike prior knowledge-based support vector machine approaches which require rules expressed as polyhedral sets, KBBN directly incorporates the rules without any modification. KBBN uses data to refine rule-based classifiers when the rule set is incomplete or ambiguous. We develop a predictive KBBN model for 69 MTBC clades found in the SITVIT international collection. We validate the approach using two testbeds that model knowledge of the MTBC obtained from two different experts and large DNA fingerprint databases to predict MTBC genetic clades and sublineages. These models represent strains of MTBC using high-throughput biomarkers called spacer oligonucleotide types (spoligotypes), since these are routinely gathered from MTBC isolates of tuberculosis (TB) patients. Results show that incorporating rules into problems can drastically increase classification accuracy if data alone are insufficient. The SITVIT KBBN is publicly available for use on the World Wide Web.

## 1. Introduction

Tuberculosis (TB) represents a reemerging serious health threat worldwide. TB is caused by the* Mycobacterium tuberculosis* complex (MTBC) bacterium. One-third of the world population is latently or actively infected with TB. Molecular epidemiology now plays a crucial role in the tracking and control of TB [1]. DNA fingerprinting methods have made it possible to distinguish between cases of recent transmission of TB and reactivation of latent infections. This has enabled the tracking of transmission routes and the timely identification of outbreaks. Thus, knowledge about the genotypes of prevailing strains has revolutionized traditional approaches to the epidemiology of TB. Moreover, the predominance of certain strains or groups of strains in certain host populations has been clearly observed [2]. Studies of the genetic and biogeographic diversity of the MTBC have revealed differences in the virulence, immunogenicity, and drug resistance of strains [2]. This has consequences for the development of control measures for TB.

**Figure 1:**
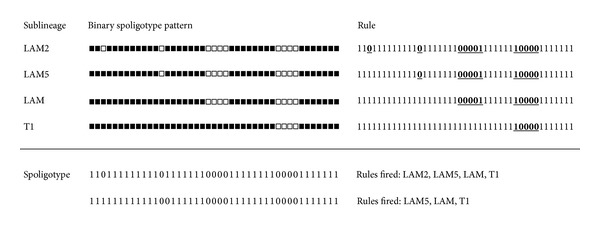
Example rules from SpolDB4. The rule column represents characteristic patterns specified by the visual rules as underlined subsequences in the spoligotype patterns. Each line corresponds to a rule. The underlined portions of the spoligotype must match exactly while the portions not underlined can take any value. All of these rules fire for the spoligotype 1101111111110111111100001111111100001111111, while three of the rules fire for 1111111111110011111100001111111100001111111.

The classification of MTBC strains into genetic groups or clades is important to help track transmission patterns and develop a better understanding of pathologic specificities in TB. Phylogeographic clades have been defined based on genetic similarities between strains and observed associations between groups of similar MTBC genotypes with host populations [3]. A variety of molecular techniques including the analysis of phylogenetically informative single nucleotide polymorphisms (SNPs) and long sequence polymorphisms (LSPs) are used to genotype MTBC strains [4]. Classification based on SNPs and LSPs is considered to be the gold standard. However, studies of such variations in DNA sequences of MTBC strains are not performed frequently for public health purposes. Spacer oligonucleotide typing (spoligotyping) and mycobacterial interspersed repetitive units-variable number of tandem repeats (MIRU-VNTRs) typing are two polymerase-chain-reaction- (PCR-) based DNA fingerprinting methods routinely used in the United States for genotyping all identified culture-positive TB cases. Large databases of spoligotypes have been collected. Each spoligotype for a strain is determined by the presence or absence of 43 specific spacers in the DR region, producing a 43-bit number. Each spacer separates two direct repeats. These strains have been assigned sublineage labels using mixed expert-based and bioinformatics approaches derived from visual rules applied to spoligotypes as shown in Figure 1.

These visual rules are based on the identification of characteristic deletions of one or more adjacent spacers in spoligotypes. Certain inferred mutations (deletions of blocks of adjacent spacers) in progenitor strains are considered to be lineage defining [5]. These deletions are conserved in all descendent strains since studies have shown that the mechanism of mutation observed in the direct repeat (DR) region involves loss of spacers, and spacers are rarely gained [6]. Additionally, the existence of these sublineages has been independently verified by clustering based on spoligotype and MIRU types of strains [7, 8]. Therefore, while it has been established that strains of TB belong to distinct sublineages, the definitions of these sublineages based on spoligotypes are not clear. The visual rules for a sublineage are generalizations of spoligotype patterns that belong to the sublineage. However, directly applying visual rules to spoligotype patterns can lead to multiple assignments of sublineage labels since spoligotype patterns may match patterns prescribed by more than one rule, and sometimes spoligotype patterns do not exactly match the patterns specified by any rule. This is an inherent limitation of a rule-based system, wherein rules need to be broad enough to capture general patterns but narrow enough to delineate classes. Additionally, spoligotyping is based on polymorphisms in a single locus, the DR region, and therefore has the potential for convergent evolution. Relying on specific subsequences within the spoligotypes for the study of genetic diversity is hence error prone.

This paper presents a hierarchical probabilistic graphical model, the knowledge-based Bayesian network (KBBN), that encodes rules of thumb and large training databases to classify data into given classes. Expert knowledge is modeled in the top level of variables in the BN representing the rules. The middle level variables represent the class and the lower level represents various features of interest. KBBN uses the strategy of not directly modeling the dependency of the rules on the features. This greatly reduces the model parameter space which helps reduce the amount of data required for training while capturing the knowledge in the rules.

The overall goal of this paper is to construct a model for predicting clades based on spoligotypes as determined by SITVIT using published rules from SPOLDB4 and other sources and to make this model available via the World Wide Web. For MTBC clade classification in this model, the visual rules of thumb are the top-level variables, the clades are the classes, and the 43 spacers that constitute the spoligotypes are the features. The KBBN for MTBC sublineages builds on the conformal Bayesian network previously designed for that domain. The structure of the KBBN encodes the knowledge base captured in the rules of thumb helping to improve overall accuracy while overcoming any potential problems such as ambiguous, inaccurate, or incomplete rules. A secondary goal is to assess the effectiveness of the KBBN in the MTBC lineage classification task. Thus we do extensive experiments on SITVIT as well as an additional testbed: CDC. The CDC test set consists of data and rules from the United States Centers for Disease Control and Prevention.

This paper is organized as follows. We first examine prior Bayesian networks for MTBC classification and then introduce the KBBN approach to incorporate rules. We then examine the rules of thumb and data associated with MTBC clades. We present results for the KBBN-SITVIT model and assess its accuracy. Finally, computational studies examine how KBBN can improve accuracy over Bayesian networks on the two KBBN testbeds (SITVIT and CDC). The results show that KBBN is quite resilient to incomplete, inaccurate, or ambiguous rules and can obtain better performance than BN using less data.

Previously in the MTBC domain, other approaches to incorporate advice in the form of rules have been shown to improve discriminative learning models of MTBC major lineages and other problems [9]. However, those methods are limited to rules expressed in less-intuitive polyhedral form that requires preprocessing of data and rules.

The proposed KBBN model allows the existing rules of thumb to be incorporated with no modification resulting in improved classification over the predictions made with the rules or Bayesian networks alone. Also, unlike visual rules, the flexibility offered by the KBBN enables it to handle these common problems with the following rules of thumb.

- Incompleteness: rules only exist for some of the classes or only partially cover a class.
- Ambiguity: multiple rules of thumb for different classes apply to the same exemplar. This frequently occurs if there is no precedence associated with the rules.
- Inaccuracy: rules may incorrectly classify some exemplars.

Visual rules with precedence have been established for six major MTBC lineages [10]. A prior online knowledge-based support vector machine (SVM) approach combined these visual rules and precedence into a set of rules expressed in polyhedral form [9]. The method produced a high-accuracy SVM using much less data. However, as discussed in Section 5, this elegant work has several practical limitations that we sought to overcome in this study. First, expressing rules and precedence as polyhedral rules [9] can be challenging for a large number of rules. Second, the method works best with linear SVMs, but linear SVMs do not capture the underlying complexity of the biomarkers and their mechanism of evolution. This can be overcome by using nonlinear SVMs (SVMs using 3-degree polynomial kernels work very well), but then incorporating the polyhedral rules becomes even more challenging. Third, the complexity of training increases with the introduction of rules. Thus, the proposed design of the KBBN has the following salient features:

- incorporates rules easily without modification and without imposing precedence,
- models known properties of the domain such as biomarkers and their mutation mechanisms,
- provides an efficient training method for classes with and without rules,
- achieves high prediction accuracy,
- overcomes ambiguity, incompleteness, and inaccuracy of the rules,
- provides additional information about the effectiveness of each rule.

The overall approach produces a high quality model for predicting SITVIT clades which has been made available for use by other researchers.

## 2. Bayesian Network Background

A Bayesian network (BN) is a graphical representation of a probability distribution. Formally speaking, a BN is a directed acyclic graph *G*(*N*, *E*) consisting of a set of nodes *X* = {*x*_*i*_ | *x*_*i*_ ∈ *N*} to represent the variables and a set of directed links to connect pairs of nodes [11]. Each node has a conditional probability distribution that quantifies the probabilistic relation between the node and its parents such that for a network of *k* nodes

(1) $$\begin{array}{c} P\left(x_{1},x_{2},\ldots ,x_{k}\right)=\overset{k}{\underset{i=1}{\prod }}P\left(x_{i}\;|\;\text{parents}\left(x_{i}\right)\right). \end{array}$$

Therefore, one can compute the full joint probability distribution from the information in the network. In other words, a well-represented Bayesian network can capture the complete nature of the relationship among a set of variables.

The SPOTCLUST Bayesian network was the first generative model used for analysis of MTBC sublineages [7]. SPOTCLUST uses mixture models based on spoligotypes to identify strain families of MTBC. SPOTCLUST models the asymmetric evolution of spacers using a Bayesian network with “hidden parents” [7]. The hidden parents of a lineage generate the members of the lineage. They capture the evolution of spoligotypes without generating the full phylogeny. A spacer in the hidden parent may be lost with small probability. A spacer that is absent in the parent is almost never gained. The design models the evolution mechanism of the DR region, allowing the Bayesian network to capture the deletions that are known to characterize spoligotype lineages. The hidden parent technique of SPOTCLUST is used for the spoligotype-associated parts of the KBBN model.

**Figure 2:**
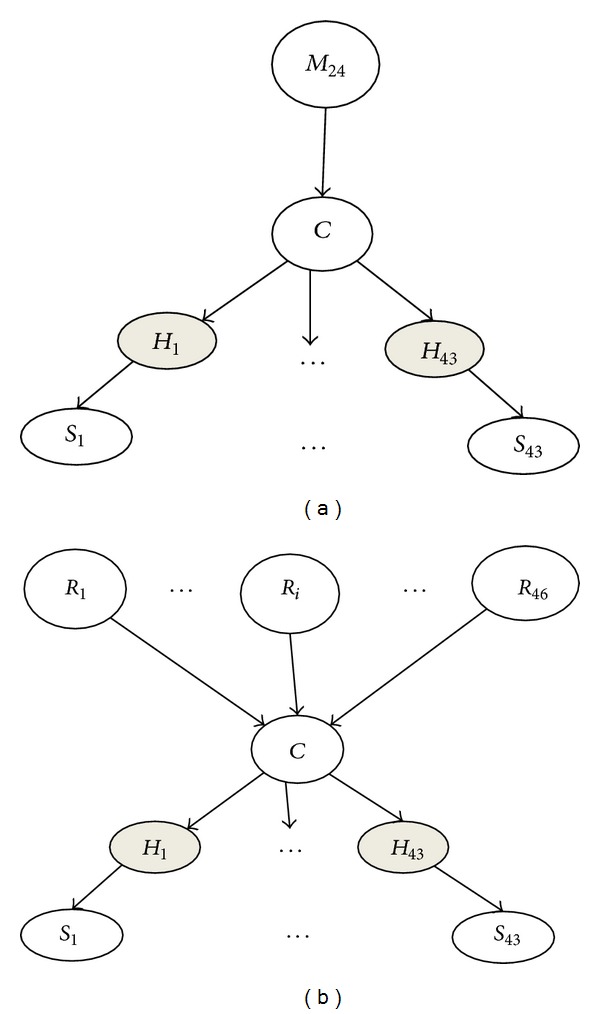
(a) The spoligotype conformal Bayesian network uses a single rule based on the number of repeats at the MIRU24 locus as the first level of a hierarchical Bayesian network. It uses the 43 spacers as features. CBN predicts the major lineage with high accuracy. (b) The KBBN uses multiple rules based on the presence of characteristic deletions as the first level of a hierarchical Bayesian network. As with the CBN, it uses the 43 spoligotype spacers.

The conformal Bayesian network (CBN) is another generative model for analysis of both spoligotype and MIRU type data for MTBC strains [9, 12] (spoligotype CBN is shown in Figure 2(a)) originally designed for predicting major MTBC lineages. CBN captures the domain knowledge about the properties of spoligotypes and MIRU and uses this information to classify MTBC strain genotyping data into major lineages. CBN reflects the known mutation mechanisms of the spoligotypes and MIRU. With rare exceptions, ancestral strains have 2 or more repeats at MIRU24. Thus the top-level variable, *M*_24_, indicates whether MIRU24 is less than two (indicating one of the modern lineages with high probability) or at least two (indicating one of the ancestral lineages with high probability).

One can think of the MIRU CBN model “generating” the data as follows. The value of locus MIRU24 generates the lineage, which in turn determines the number of repeats in the remaining MIRU loci. Thus, patterns in the occurrences of repeats at each locus for each lineage are captured. The lineage also generates the hidden parents of the lineage which in turn generate the spoligotype spacers. The MIRU24 determines the lineage priors.

We tried using the CBN model as designed for major lineages to classify MTBC genotyping data into sublineages. But using the single rule,* if MIRU*24 ≥ 2*, then lineage is ancestral*, as in the original CBN was not enough to generate a good model. KBBN grew out of the effort to incorporate all of the visual rules available from SpolDB4 [3] into CBN.

### 2.1. Knowledge-Based Bayesian Network

The knowledge-based Bayesian network (KBBN) is a hierarchical probabilistic graphical model which encodes the knowledge obtained from expert-defined rules derived from true observations with large databases to solve classification problems. KBBN incorporates the rules of thumb as high level variables or class priors in the Bayesian network and therefore combines the information obtained from rules of thumb with the information provided by a BN model specifically designed for the domain. The method is designed and tested on widely used simple BNs such as naïve Bayes and polytrees with polynomial time learning and inference algorithms. The KBBN for the tuberculosis domain uses CBN as its base and is used for both CDC and SITVIT testbeds.

KBBN, represented in Figure 2(b), is a novel hierarchical Bayesian network probability model for sublineage classification of MTBC. KBBN captures domain knowledge about the properties of spoligotypes and incorporates additional information provided by SpolDB4 or CDC rules to predict the class with high accuracy. The corresponding probability density function for the naïve KBBN model, shown in Figure 2(b), is

(2) P(C,SΩ,RΨ)=∏j∈Ω(∑Hj∈{0,1}P(Sj ∣ Hj)P(Hj ∣ C))×P(C ∣ RΨ)P(RΨ),

where the random variable *C* represents the sublineage class, the random variable *S*_*Ω*_ = {*S*_*j*_ | *j* ∈ *Ω*} with *Ω* = {1,…, 43} represents the spoligotype spacers, and *R*_Ψ_ = {*R*_*k*_ | *k* ∈ Ψ} represents the set of binary rules indicating whether each specific rule is fired. The spacer variables *S* and class variable *C* are assumed to follow binomial and multinomial distributions, respectively. The conditional probabilities of *R* given *C* are represented as a table which maps the set of possible combinations of rules fired in the data to the probability of each class. Laplacian smoothing is used.

For spoligotypes, we followed the SPOTCLUST model [7]. It captures the fact that spacers are lost but almost never gained, by introducing a variable for the unobserved hidden parent (*H*_*j*_) and for each spacer *S*_*j*_, both of which follow a binomial distribution. Given a 43-dimensional spoligotype *S* and its spacer position *j*, *S*_*j*_ = 1 if the spacer is present, and *S*_*j*_ = 0 if the spacer is absent. The probabilities of the spacer given the parent *P*(*S*_*j*_ | *H*_*j*_) are assumed to be known. As in Vitol et al., 2006 [7], we considered the probability of losing a spacer as 10^−1^ and the probability of gaining a spacer as 10^−7^.

The KBBN assumes that the spoligotype hidden parents are conditionally independent given the sublineage. The conditional independence assumption of spacers is a model simplification previously made in the SPOTCLUST BN model. This conditional independence of the biomarkers in the BN model enables KBBN to conform to the set of available biomarkers without any expensive missing value computations. None of the genotyping variables in the BN are treated as unobserved except for the hidden parent spacers, which are always unobserved.

Using Bayes' rule, one can predict the sublineage for new data by determining the sublineage with maximum probability:

(3) P(C ∣ SΩ,RΨ)∝∏j∈Ω(∑Hj∈{0,1}P(Sj ∣ Hj)P(Hj ∣ C))×P(C ∣ RΨ)P(RΨ).

## 3. Data Domains and Biology Rules 

This study focused on creating a predictive model for clades that emulated SITVITWEB, a publicly available international multimarker database for tuberculosis molecular epidemiology [13]. Different datasets were created for training, testing, and cross validation. To validate the approach we also used a dataset of isolates collected by the CDC for cross validation studies. The following two sections describe the datasets in detail.  Table 1 summarizes them.

### 3.1. SITVIT Testbed

SITVIT-Train and SITVIT-Test are based on the SITVIT, a MTBC genotyping markers database provided by the Institut Pasteur de la Guadeloupe, and on the SpolDB4 rules that are published in Brudey et al., 2006 [3], plus one rule recently developed for the URAL1 clade. KBBN was trained on the SITVIT-Train dataset of 2714 records, each corresponding to a spoligotype and clade. There were 69 classes, the minimum sublineage size was 1, and the maximum sublineage size was 390. To test this model while keeping all classes we used SITVIT-Test, a large dataset based on SITVIT with 7949 records, each record corresponding to a spoligotype and clade. This dataset contained the same 69 classes as SITVIT-Train with different class distributions and again with the minimum class size of 1. SITVIT-Train and SITVIT-Test do not overlap so the total SITVIT dataset consists of 10633 distinct spoligotypes. To enable 10-fold cross validation (CV) with at least one spoligotype per class, the SITVIT-CV dataset was created which consists of the SITVIT-Train data restricted to the 45 classes with at least 11 spoligotypes each.

Note that some lineages have been reclassified while the KBBN model was under development. Two LAM sublineages were recently raised to lineage level: LAM10-CAM as the Cameroon lineage [14] and LAM7-TUR as the Turkey lineage [15, 16]. Some spoligotype patterns previously classified as H3 and H4 sublineages were relabeled “Ural” [17]. The latter include patterns belonging to H4 sublineage that were relabeled “Ural-2” and some patterns previously classified as H3 sublineage but with an additional specific signature (presence of spacer 2, absence of spacers 29 to 31 and 33 to 36), which are now relabeled “Ural-1.” With their definitive reclassification pending, we hereby refer to these as H4-Ural-2, H3, and H3-Ural-1. Spoligotype patterns labeled as EAI and EAI5 were merged into a single group called EAI since one rule covers both patterns.

A sample of SpolDB4 rules is presented in Figure 1. Each line corresponds to a rule. The underlined portions of the spoligotype must match exactly while the portions not underlined can take any value. Note that in Brudey et al. [3] the rules are expressed using the octal coding of spoligotypes; here we express them in binary for simplicity. While these rules establish characteristic patterns for sublineages of MTBC, they are not exclusive and in some cases overlap. Up to 4 rules fired per example. The mode of the number of rules fired per record was 2. In practice, a precedence or order is introduced over the rules using expert knowledge so that unambiguous sublineage predictions are generated. However, this precedence has not been published for sublineages and is up to the individual user of the rules. The SpolDB4 rules have continued to evolve as new lineages such as H3-URAL-1 which are added and refined, and thus the exact rules that we used are provided in Supplement 1 in the Supplementary Materials available online at http://dx.doi.org/10.1155/2014/398484.

### 3.2. CDC-Sublineage and CDC Rules

The second dataset, CDC-Sublineage, examines 1286 MTBC isolates genotyped by spoligotyping and labeled with 8 sublineages. Dr. Lauren Cowan of the CDC was interviewed to obtain 8 rules of thumb. The data is a subset of 31,482 MTBC isolates genotyped by spoligotyping and 12-locus mycobacterial interspersed repetitive units (MIRU) typing with known lineages from a set collected by the CDC as part of routine TB surveillance in the United States from 2004 to 2009. Since only spoligotypes are used in the rules, the data for training were restricted to spoligotypes with labeled sublineages.

There are 8 rules expressed as numeric formulas based on the 43 spacers in the spoligotypes. For example, the rule for the Indo-Oceanic sublineage Manila (or EAI2-Manila in SpolDB4 rules) is

 If absence of spacers 3, 20, 21, 29–32, 34, sum(spacers 33–36) > 0 and presence of spacers 2, 4, 19, 22, then Indo-Oceanic Manila.

There is one rule per sublineage. This dataset was preprocessed by adding an array of 8 bits, one bit per rule. The value of a bit was set to 1 if the rule was fired and zero otherwise. Note that the sublineage sizes are unequal. Overall, the minimum sublineage size was 39, the maximum sublineage size was 356, and the median was 138 records. The rules were ambiguous and no precedence was imposed. In some cases no rules fire for a record. A maximum of 2 rules is fired for each record. The mode of the number of rules fired per record was also 2. If multiple rules fire for a record and the sublineages determined conflict or if no rules fire, the record is considered to be misclassified. Details of the CDC rules can be found in Supplement 2.

## 4. SITVIT Experimental Results

In this section, we examine the effectiveness of the KBBN model for prediction of SITVIT classification results. Our experiments consist of two parts: (1) in-sample accuracy of the SITVIT KBBN model trained using all available data and (2) out-of-sample accuracy of the SITVIT KBBN model trained on the SITVIT-Train and tested on the much larger SITVIT-Test set.The accuracy of the results was measured using the *F*-measure on the testing data (harmonic mean of precision and recall) averaged over the classes. The *F*-measure was selected since it effectively captures performance on the unbalanced multiclass data sets studied here. Reporting class accuracies/errors can be misleading for unbalanced classes such as those in the TB data. The minimum and maximum class sizes are reported in Table 1. The *F*-value was computed as

(4) $$\begin{array}{c} F=2\times \frac{\text{precision}\times \text{recall}}{\text{precision}+\text{recall}}, \end{array}$$

where recall is the percentage of the isolates in a given clade correctly identified as being in that clade and precision is the percentage of isolates predicted to be in a clade that are actually in the clade.

### 4.1. SITVIT KBBN Model Accuracy

The SITVIT KBBN model was trained to predict 69 sublineages using the combined SITVIT-Train and SITVIT-Test data extracted from SITVITWEB along with the SPOLDB4 rules. Overall the model is very accurate; it correctly classifies 94.3% of all of the spoligotypes, achieving an average *F*-value of 0.93 across the 69 clades.  Table 2 presents the in-sample results of SITVIT-KBBN for each clade. The errors that do occur primarily come from lack of specificity not sensitivity. The model achieves a sensitivity of greater than 82% on all of the clades, but the specificity is below 82% on 13 clades. The T clade, which is known to be ill defined, contributes errors leading to reduced specificity in a wide variety of clades including LAM6, T1-RUS, T3-ETH, T3-OSA, and AFRI. Within the* M. africanum* clades, AFRI is primarily confused with other* M. africanum* clades (AFRI_1, AFRI_2, and AFRI_3) which is an acceptable error. A few Cameroons, H3, and T isolates are mistakenly identified as AFRI. Many BOV isolates are assigned by the model as belonging to BOV_3 indicating that a more expansive definition of BOV_3 may be warranted. There are some minor confusions within the Haarlem sublineages H1, H2, and H3 combined with the new H4-URAL-2 and H3-URAL-1 sublineages. About 16% of H3 is assigned to other classes. This suggests that further refinement of the definition of these sublineages will be ongoing. Microti, PINI, and PINI2 have lower *F*-values, but this is partially due to the fact that these sublineages have only a few exemplars. More data is needed for these rare lineages to improve the model. The *F*-value of ZERO is reduced by 6 CAS misclassified as ZERO. The overall specificity averaged over the clades is 0.909 and the sensitivity is 0.965.

### 4.2. Predictive Accuracy Results

To assess the out-of-sample predictive accuracy of the KBBN SITVIT model we trained the model on SITVIT-Train and tested it on SITVIT-Test. The model was very accurate overall achieving an average out-of-sample test *F*-value of 0.939, almost identical to the in-sample estimate of above 0.930. The average recall (percentage of the isolates in a given clade correctly identified as being in that clade) between all lineages is 97.5%, and the average precision (the percentage of isolates predicted to be in a clade that are actually in the clade) among all lineages is 91.9%. As shown in Table 3, the results for each clade are very similar to those reported in Table 2. The T clade and small rarer clades such as PINI variants and Microti account for much of the decrease in precision.

### 4.3. Model Validation

The next set of experiments evaluates the effectiveness of the KBBN approach with respect to other techniques and the effectiveness of incorporating rules. All experiments were done on both the SITVIT and CDC datasets to ensure that the results are not an artifact of a single dataset. For each dataset, first we used 10-fold stratified cross validation. Each training set was divided into 10 parts with 9 parts available as the training data for creation of models and 1 part held out as an independent test set. For all experiments the same test sets were employed, but the training dataset or the set of rules used may be varied. The accuracy of the results was measured using the *F*-measure on the testing data (harmonic mean of precision and recall) averaged over the classes. To facilitate a fair comparison, the data were constructed so that there are at least 10 records per class. In the SITVIT domain, this required removing clades that do not commonly infect human beings (e.g., PINI1 and PINI2). We refer to this subset of the SITVIT-Train dataset as SITVIT-CV. SITVIT-CV had 45 classes and 2593 records. The minimum sublineage size was 11, and the maximum sublineage size was 390 with a mode of 21 records.

#### 4.3.1. Comparison with Other Techniques

We designed several sets of experiments on the two datasets SITVIT-CV and CDC-Sublineage to determine the following: if incorporating rules improved the performance of the Bayesian network over the performance of the BN or rules alone. The results were gathered for KBBN, BN, and the rules used alone. In addition, linear and nonlinear SVM results were provided as a baseline for comparison. The SVM implementation in WEKA (http://www.cs.waikato.ac.nz/ml/weka/) was used. The SVM kernels and parameters were selected using a grid search of 9-fold cross validated accuracy of the training set. The degree-three polynomial kernel and radial basis function kernels were found to work best. All SVM data was normalized before training. Also, we are interested in the nature of the misclassification because it tells us about the potential inaccuracies in the definition of the lineages.

Table 4 compares the results of KBBN, BN, Rules-only, and SVM (nonlinear and linear) on the two testbeds. The rules themselves have very poor overall accuracy, but they led to improvements over the baseline BN accuracy on both datasets with statistically significant improvements on CDC-Sublineage and SITVIT-CV. The SVM results indicate that KBBN's accuracy is competitive with state-of-the-art nonlinear and linear classification methods. But note that KBBN, being a generative method, has many advantages over SVM such as availability of posterior probabilities of each class given the observation that can be interpreted as the confidence of the prediction, easier interpretation, and ease of incorporation of domain knowledge.

#### 4.3.2. Effectiveness of Rules in Bayesian Network

Next, we designed several sets of experiments to determine the following: how the quality and quantity of rules and data affected the performance of KBBN. The basic underlying experimental design was the same for experiments across the two testbeds.

**Figure 3:**
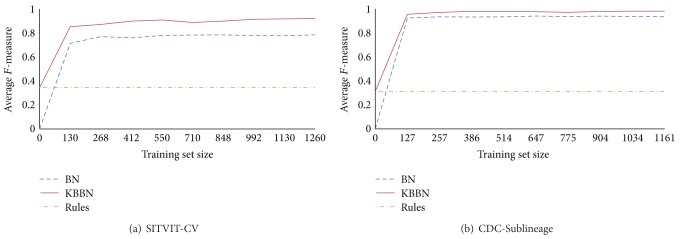
The result of adding rules to different training set sizes for the (a) SITVIT-CV and (b) CDC-Sublineage testbeds.

Our hypothesis was that KBBN can learn the concept faster with less data by adding rules. We wanted to show that rules can improve learning especially where you have less data. For each dataset, first we used 10-fold stratified cross validation. Next each training set was divided into 9 parts providing models using 1/9, 2/9,… or 9/9 of the training set and tested on the corresponding test set. The test sets were kept the same for different training set sizes. We measured the amount of *F*-value for different training set sizes with or without the rules and compared the result with the case of using no data at all (i.e., BN case) or only rules. The results are presented in Figure 3. Similar smaller testing set studies on CDC-Sublineage and SITVIT-CV found that KBBN always performs better than or as good as BN for all training set sizes.

To further examine the effect of incorporating rule sets and using incomplete rules, we performed two sets of experiments described in the following section: (1) using increasing percentages of the available rules and (2) using subsets of rules, removing rules for a given class at a time.

#### 4.3.3. Removal of Rule Sets for a Class 

**Figure 4:**
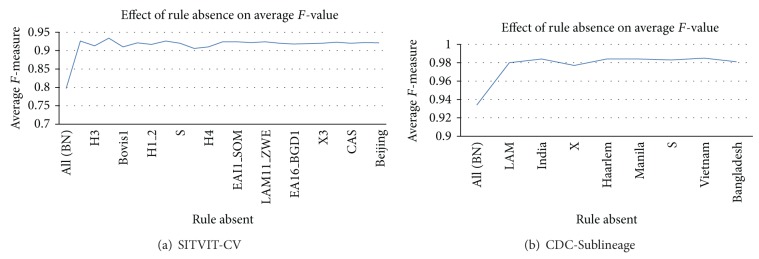
Effect of removing rules for each class on the average *F*-value for (a) SITVIT-CV and (b) CDC-Sublineage.

In these experiments, we examined the effect of removing all the rules associated with a given class. We examined the KBBN accuracy and recorded the amount of average *F*-value between all classes after all the rules corresponding to a single class are removed. Again, 10-fold stratified cross validation was performed. The results are presented in Figure 4. “All (BN)” is when no rules are used in KBBN, which is equivalent to BN performance. Clearly, KBBN can lead to significant improvements compared to when no rules exist for entire classes of MTBC. We leave a more comprehensive study of when rules are most helpful for problems in other domains to future work.

## 5. Quality of Rules

KBBN can provide us with information about the quality of each rule. We studied posterior probabilities of rules given the class to provide insight into the utility and accuracy of each rule. The *P*(*r* | *c*) is of great interest because it tells us how good rule *r* is for a given class *c*. The posterior probability of the rules given the classes for the CDC-Sublineage data is presented in Table 5. The table includes a row for “No rule” indicating the probability of no rule getting fired. When no rule is fired a regular BN is used instead of KBBN. Note that the probabilities within columns may sum to more than 1 since rules are not mutually exclusive.

For CDC, the rule for LAM exactly corresponds to the class LAM on this data, since *P*(Rule = LAM | Class = LAM) = 1 and all other probabilities in the LAM row or column are 0. The rules for S and X correctly fire for their respective classes, but they also fire incorrectly for other lineages as indicated by the other entries in the S and X rows. The rules for Haarlem and Manila correctly predict their corresponding sublineages, but the fact that “No Rule” occurs 29.6% and 24.3% of the time, respectively, indicates that these rules fail to cover all members of their class. For the India class, the India rule is quite accurate, but the rules can be ambiguous as indicated by the multiple entries in the India column. Most Vietnams are not covered by any rules and for those that are covered the rules may be ambiguous.

**Figure 5:**
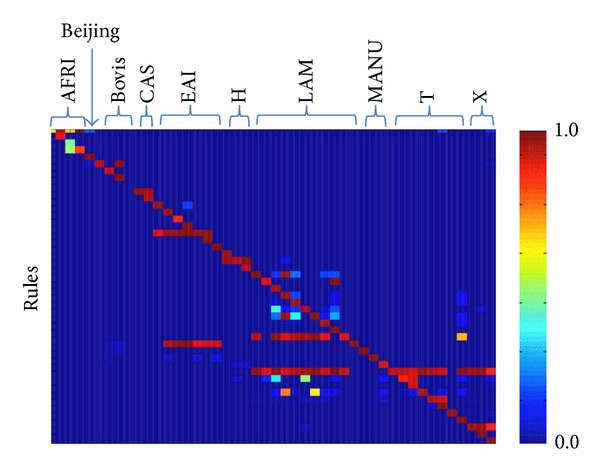
The heat map represents the posterior probability of each rule given the sublineage for the SITVIT dataset. A strong association of a rule in predicting a sublineage is shown with a red square while a blue square represents no relation. Here H includes URAL-1 and URAL-2 and LAM includes Turkey and Cameroon sublineages.

We provide the posterior probability distribution of each rule given the sublineage for the SITVIT-CV dataset as a heat map in Figure 5. Good rules only have red on the diagonal. A rule fires for multiple classes if it has multiple red entries in a row. The rule set is ambiguous for a class if there are multiple red entries within a given class column. Notice that the rules that are fired for many classes with high probability (e.g. T) are not very effective in indicating the associated class as opposed to Beijing which is an effective rule.

## 6. Discussion of Alternative Knowledge-Based Approaches

The KBBN has a great appeal over alternative knowledge-based approaches such as knowledge-based SVM (KBSVM) and knowledge-based neural networks (KBANN) [8, 9, 18]. The first advantage is that no special encodings of the rules are required. In KBANN, the rules are mapped into a neural network by converting the data to numeric form and designing appropriate nodes, links, and weights in the neural network. KBSVM requires each rule to be encoded as a polyhedral rule, such as *if* *x* ∈ *R* ^*n*^ *satisfies* *Bx* ≤ *d* *then* *class* = 1. In KBSVM, the process of converting rules to probabilities can greatly increase the number of rules. For example, for the task of predicting major lineages of MTBC, the 13 original logical rules published in a prior study [8] were mapped into 29 polyhedral rules. The added rules help capture the precedence of the original rules which made them mutually exclusive. There is no easy way to capture rule precedence in KBSVM or KBANN. KBSVM must add rules of the form “*if* *x* *satisfies* *condition* *A* *then* *x* *is* *not* *in* *Class* *y*.” In KBBN, the data can be numeric or symbolic and each rule may be any arbitrary function of the observations to the classes. As reported in the preliminary study [9], KBBN works effectively on rules with and without precedence.

A second advantage of KBBN is the computational complexity of training. For the polytree type KBBN studied here, both training and inference can be done very efficiently in polynomial time. The MAP estimation of KBBN parameters has a closed form solution enabling globally optimal solutions to be found by simple counting algorithms. The only additional computation required over BN is representation of the probabilities of the classes given the rules. Unlike KBSVM and KBANN, no special purpose software is required for mapping and training KBBN beyond knowing which rules are fired for which example. KBSVM requires the solution of challenging nonconvex programs with many constraints and variables introduced for each rule over the original SVM. Similarly, KBANN also requires the solution of a nonconvex program of considerably greater complexity than the original ANN network due to the addition of weights and nodes to capture the rules. Special purpose software is needed to create the structure of the KBANN network, but any neural network training algorithm can be used to train it.

Additional benefits of KBBN over KBSVM and KBANN include that KBBN can be easily used for multiclass problems, it provides estimates of the posterior probabilities of the classes, and the resulting classification function is more transparent and explainable. KBSVM classification results published to date are limited to two-class problems and how to do efficient multiclass KBSVM remains an open research question. In both KBSVM and KBANN, the rules are used only to bias the construction of the prediction function and the prediction is typically a black box. In contrast, the KBBN probability density functions are readily interpretable as soft relaxations of the visual rules already used in TB. The posterior probabilities of the rules can be used to explain the effectiveness of these rules.

## 7. Conclusions 

We have developed an effective classifier to predict SITVIT MTBC clades with high accuracy. The result is a publicly available web-based tool for SITVIT clade classification to support TB control and research efforts available for use at TB-INSIGHT (http://tbinsight.cs.rpi.edu/run_tb_lineage.html) and later on SITVIT2. We established that the clade estimates are robust by performing two out-of-sample testing experiments. Furthermore, the results on the two testbeds show that KBBN is a highly accurate classifier that can outperform methods based on rules or Bayesian networks trained on data alone and that meets or beats the performance of nonlinear and linear SVM models. KBBN proved to be robust to ambiguity, incompleteness, and inaccuracy of the rule set. The results here are limited to simple commonly used BN that are polytrees using MAP estimation, but future work is needed to examine the KBBN approach on more general BN models and algorithms.

As a general approach, KBBN has many attractive properties. It allows any type of rules to be incorporated into a Bayesian network with little increase in the model and training complexity. Prior knowledge-based SVM required manipulation of the rules, models, data, and/or kernel [8, 9]. There is no need to introduce precedence or resolve inconsistency of the rules for KBBN. The KBBN model can provide posterior class probabilities as well as information on how the rules were used and how classification decisions were made. We studied the posterior probability of rules given the class to provide insight into the utility of each rule. This underlines another advantage of KBBN as a generative model over its discriminative competitor models, like KBSVM. Thus KBBN offers a promising research direction for combining rule and data-driven predictive methods that may be applicable in many domains.

## Supplementary Material

Supplement 1: SITVIT Rules used in KBBN. The following rules, based on presence and absence of spacers in a spoligotype, are used to determine the SITVIT clade.
Supplement 2: CDC Rules used in KBBN. The following rules, based on presence and absence of spacers in a spoligotype, are used to determine the CDC sublineage. The notation sum(sp33-36) > 0 means that the sum of spacers 3 to 36 are greater than 0. This is equivalent to saying that at least one of the spacers from 33 to 36 is greater than 0.

## Figures and Tables

**Table 1:** SITVIT and CDC MTBC testbeds.

Testbed	Dataset	Size	Number of classes	Max class size	Min class size	Number of rules
SITVIT	Train	2714	69	390	1	69
	Test	7949	69	1107	1	69
	CV	2593	45	390	11	45

CDC	CV	1286	8	356	39	8

**Table 2:** Training *F*-measure for KBBN trained on all 10633 SITVIT isolates.

Clade	*F*-measure	Clade	*F*-measure	Clade	*F*-measure
AFRI	0.800	H	0.736	PINI	0.750
AFRI_1	0.944	H1	0.924	PINI1	1.000
AFRI_2	0.908	H2	0.875	PINI2	0.667
AFRI_3	0.966	H3	0.915	S	0.976
Beijing	1.000	H3-Ural-1	0.873	T	0.926
BOV	0.948	H37Rv	0.958	T1-RUS2	0.778
BOV_1	0.993	H4-Ural-2	0.933	T2	0.953
BOV_2	1.000	LAM	0.947	T2-Uganda	0.991
BOV_3	0.644	LAM1	0.977	T3	0.964
BOV_4-Caprae	0.891	LAM11-ZWE	0.954	T3-ETH	0.65
Cameroon	0.929	LAM12-Madrid1	0.947	T3-OSA	0.626
CANETTI	1.000	LAM2	0.991	T4	0.988
CAS	0.937	LAM3	0.988	T4-CEU1	1.000
CAS1-Delhi	0.961	LAM4	0.970	T5	0.984
CAS1-Kili	0.973	LAM5	0.978	T5-Madrid2	1.000
CAS2	0.921	LAM6	0.856	T5-RUS1	0.949
EAI	0.982	LAM8	1.000	T-Tuscany	1.000
EAI1-SOM	0.986	Manu_ancestor	1.000	Turkey	0.928
EAI2-Manila	0.984	Manu1	0.991	X1	0.989
EAI2-Nonthaburi	1.000	Manu2	1.000	X2	0.963
EAI3-IND	0.963	Manu3	1.000	X3	0.995
EAI4-VNM	1.000	Microti	0.750	ZERO	0.800
EAI6-BGD1	0.989				
EAI7-BGD2	1.000			AVERAGE	**0.930**
EAI8-MDG	1.000				

**Table 3:** Results of the *F*-measures of KBBN based on out of-sample test. The KBBN model was trained on SITVIT-Train (with 2714 records) and tested on SITVIT-Test with 7949 records. Overall average *F*-measure is 0.939.

Clade	*F*-measure	Clade	*F*-measure	Clade	*F*-measure
AFRI	0.889	H	0.942	PINI	0.667
AFRI_1	0.975	H1	0.977	PINI1	0.923
AFRI_2	0.926	H2	0.625	PINI2	0.522
AFRI_3	1.000	H3	0.944	S	0.956
Beijing	0.980	H3-Ural-1	0.887	T	0.969
BOV	0.981	H37Rv	1.000	T1-RUS2	0.956
BOV_1	0.996	H4-Ural-2	0.960	T2	0.991
BOV_2	1.000	LAM	0.949	T2-Uganda	1.000
BOV_3	1.000	LAM1	0.986	T3	0.969
BOV_4-Caprae	0.914	LAM11-ZWE	0.976	T3-ETH	0.977
Cameroon	0.967	LAM12-Madrid1	1.000	T3-OSA	0.978
Canetti	0.500	LAM2	0.993	T4	0.984
CAS	0.990	LAM3	0.973	T4-CEU1	1.000
CAS1-Delhi	0.990	LAM4	0.967	T5	1.000
CAS1-Kili	0.846	LAM5	0.985	T5-Madrid2	1.000
CAS2	1.000	LAM6	0.889	T5-RUS1	0.883
EAI	0.989	LAM8	0.970	T-Tuscany	0.889
EAI1-SOM	1.000	Manu_ancestor	1.000	Turkey	0.941
EAI2-Manila	1.000	Manu1	0.995	X1	0.963
EAI2-Nonthaburi	0.933	Manu2	0.997	X2	0.944
EAI3-IND	1.000	Manu3	1.000	X3	0.971
EAI4-VNM	1.000	Microti	0.667	ZERO	0.800
EAI6-BGD1	1.000				
EAI7-BGD2	0.993			Average	**0.939 **
EAI8-MDG	1.000				

**Table 4:** Average *F*-measure of KBBN, BN, Rules-only, and SVM (nonlinear and linear) on two testbeds. While using Rules-only provides poor results, KBBN is able to provide results that are significantly better or at least not worse than BN and SVM on both domains. Results significantly different from KBBN at 5% significance level are shown in bold.

Dataset	Model				
	KBBN	BN	Rules-only	SVM nonlinear	SVM linear
SITVIT-CV	0.945	**0.771 **	**0.345 **	**0.903**	**0.914 **
CDC-Sublineage	0.981	**0.934 **	**0.312 **	0.994	0.993

**Table 5:** Posterior probability of each rule given class for CDC-Sublineage dataset. Blanks indicate 0.

	Class						
	Haarlem	LAM	S	X	India	Manila	Vietnam
Rule							
Haarlem	0.707				0.015		
LAM		1.000					
S			1.000		0.015	0.005	0.033
X				1.000	0.015		0.017
India					0.970	0.022	0.017
Manila						0.735	
Vietnam							0.283
No rule	0.297				0.015	0.243	0.700

## References

[1] de Viedma DG, Mokrousov I, Rastogi N (2011). Innovations in the molecular epidemiology of tuberculosis. *Enfermedades Infecciosas y Microbiología Clínica*.

[2] Gagneux S, DeRiemer K, Van T (2006). Variable host-pathogen compatibility in *Mycobacterium tuberculosis*. *Proceedings of the National Academy of Sciences of the United States of America*.

[3] Brudey K, Driscoll JR, Rigouts L (2006). *Mycobacterium tuberculosis* complex genetic diversity: mining the fourth international spoligotyping database (SpoIDB4) for classification, population genetics and epidemiology. *BMC Microbiology*.

[4] Filliol I, Motiwala AS, Cavatore M (2006). Global phylogeny of *Mycobacterium tuberculosis* based on single nucleotide polymorphism (SNP) analysis: insights into tuberculosis evolution, phylogenetic accuracy of other DNA fingerprinting systems, and recommendations for a minimal standard SNP set. *Journal of Bacteriology*.

[5] Warren RM, Streicher EM, Sampson SL (2002). Microevolution of the direct repeat region of *Mycobacterium tuberculosis*: implications for interpretation of spoligotyping data. *Journal of Clinical Microbiology*.

[6] Ozcaglar C, Shabbeer A, Vandenberg S, Yener B, Bennett KP (2011). Sublineage structure analysis of *Mycobacterium tuberculosis* complex strains using multiple-biomarker tensors. *BMC Genomics*.

[7] Vitol I, Driscoll J, Kreiswirth B, Kurepina N, Bennett KP (2006). Identifying *Mycobacterium tuberculosis* complex strain families using spoligotypes. *Infection, Genetics and Evolution*.

[8] Kunapuli G, Bennett KP, Shabbeer A, Maclin R, Shavlik J, Balcázar JL, Bonchi F, Gionis A, Sebag M (2010). Online knowledge-based support vector machines. *Machine Learning and Knowledge Discovery in Databases*.

[9] Aminian M, Ozcaglar C, Shabbeer A, Vandenberg S, Hadley K, Bennett KP Knowledge-based Bayesian network for the classification of *Mycobacterium tuberculosis* complex sublineages.

[10] Shabbeer A, Cowan LS, Ozcaglar C (2012). TB-Lineage: an online tool for classification and analysis of strains of *Mycobacterium tuberculosis* complex. *Infection, Genetics and Evolution*.

[11] Koller D, Friedman N (2009). *Probabilistic Graphical Models: Principles and Techniques*.

[12] Aminian M, Shabbeer A, Bennett KP (2010). A conformal Bayesian network for classification of *Mycobacterium tuberculosis* complex lineages. *BMC Bioinformatics*.

[13] Demay C, Liens B, Burguière T (2012). SITVITWEB—a publicly available international multimarker database for studying *Mycobacterium tuberculosis* genetic diversity and molecular epidemiology. *Infection, Genetics and Evolution*.

[14] Koro FK, Simo YK, Piam FF (2013). Population dynamics of tuberculous Bacilli in Cameroon as assessed by spoligotyping. *Journal of Clinical Microbiology*.

[15] Abadia E, Zhang J, dos Vultos T (2010). Resolving lineage assignation on *Mycobacterium tuberculosis* clinical isolates classified by spoligotyping with a new high-throughput 3R SNPs based method. *Infection Genetics and Evolution*.

[16] Kisa O, Tarhan G, Gunal S (2012). Distribution of spoligotyping defined genotypic lineages among drug-resistant *Mycobacterium tuberculosis* complex clinical isolates in Ankara, Turkey. *PLoS ONE*.

[17] Mokrousov I (2012). The quiet and controversial: ural family of *Mycobacterium tuberculosis*. *Infection Genetics and Evolution*.

[18] Towell G, Shavlik J (1994). Knowledge-based artificial neural networks. *Artificial Intelligence*.

