# Supplementary material for: Predicting Mycobacterium tuberculosis Complex Clades Using Knowledge-Based Bayesian Networks
Source: Biomed Res Int. 2014 Apr 23;2014:398484. doi: 10.1155/2014/398484 (PMC4016944; doi:10.1155/2014/398484)
Supplement: Supplementary file 1 — Supplement 1: SITVIT Rules used in KBBN. The following rules, based on presence and absence of spacers in a spoligotype, are used to determine the SITVIT clade. Supplement 2: CDC Rules used in KBBN. The following rules, based on presence and absence of spacers in a spoligotype, are used to determine the CDC sublineage. The notation sum(sp33-36) > 0 means that the sum of spacers 3 to 36 are greater than 0. This is equivalent to saying that at least one of the spacers from 33 to 36 is greater than 0. [file 398484.f1.pdf]

Supplement 1: SITVIT Rules used in KBBN.

The following rules, based on presence and absence of spacers in a spoligotype,

are used to determine the SITVIT clade.

No.	Description
1	If presence of spacer 40, absence of spacers 8, 9, 39, then **AFRI.**
2	If presence of spacers 6, 10, 38, 40, absence of spacers 7-9, 39, then **AFRI_1.**
3	If presence of spacers 7, 13, 20, 25, 36, 40, absence of spacers 8-12, 21-24, 37-39, then **AFRI_2.**
4	If presence of spacers 7, 13, 36, 40, absence of spacers 8-12, 37-39, then **AFRI_3.**
5	If absence of spacers 1-34, then **Beijing.**
6	If absence of spacers 3, 9, 39-43, then **BOV.**
7	If presence of spacers 2, 8, 10, 15, 17, absence of spacers 3, 9, 16, 39-42, then **BOV_1.**
8	If presence of spacers 2, 4, 5, 7, 13-15,17, absence of spacers 3, 6, 8-12, 16, 39-43, then **BOV_2.**
9	If presence of spacers2, 4, 15, 17, absence of spacers 3, 5-14, 16, 39-43, then **BOV_3.**
10	If absence of spacers 1, 3, 16, 28, 39-43, then **BOV_4-Caprae.**
11	If presence of spacer 3, absence of spacers 4-7, 23 to 34, 20-22 35, then **CAS.**
12	If presence of spacers 3, 8, 22, 35, absence of spacers 4-7, 23-34, then **CAS1-Delhi**.
13	If presence of spacers 8-9, 11-12, 19 absence of spacers 4-7, 20-35, then **CAS1-Kili**.

14	If presence of spacers 3, 22, 35, absence of spacers 4-10, 23-34, then **CAS2.**
15	If presence of spacers 33, 39 and 41, absence of spacers 29-32, 34, 40, then **EAI1-SOM.**
16	If presence of spacers 2, 4, 19, 22, 33, absence of spacers 3, 20-21, 29-32, 34, then **EAI2-Manila**.
17	If presence of spacers 1, 2, 4, 33, absence of spacer 3, 8 – 25, 29-32, 34, then **EAI2-Nonthaburi**.
18	If presence of spacers 33, 36, 40, absence of spacers 2, 3, 29-32, 37-39, then **EAI3-IND**.
19	If presence of spacers 25, 28, 33, absence of spacers 26-27, 29-32, 34, then **EAI4-VNM.**
20	If presence of spacer 33, absence of 29-32, 34, then **EAI**.
21	If presence of spacers 22, 28, 33, absence of spacers 23, 29-32, 34, then **EAI6-BGD1**.
22	If presence of 24, 35-36, 38, absence of 25-34, 37, then **EAI7-BGD2.**
23	If presence of spacers 4, 18, 20, 33, absence of spacers 2-3, 19, 29-32, 34, then **EAI8-MDG.**
24	If absence of 31, 33-36, presence of spacer 32, then **H.**
25	If absence of 26-31 and 33-36, presence of 25, 32, then **H1**.
26	If presence of spacers 25, 32, absence of spacers 1-24, 26-31, 33-36, then **H2.**
27	If presence of spacer32, absence of spacer 31, 33-36, then **H3**.
28	If presence of spacers 2, 28, 32, absence of spacers 29 - 31, 33 – 36, then **H3-URAL-1**
29	If presence of spacers 19, 22, 31-32, absence of spacers 20-21, 33-36, then **H37Rv.**
30	If presence of spacers 28, 32, absence of spacers 2, 29-31, 33-36, then **H4-URAL-2**.
31	If absence of spacers 21-24, 33-36, then **LAM.**
32	If presence of 25, 31, absence 3, 21-24, 33-36, then **LAM1.**

33	If presence of spacer 22, 26, 31, absence of spacers 23-25, 33-36, then **Cameroon.**
34	If presence of spacers 25, 26, 31, 32, absence of spacers 21-24, 27-30, 33-36, then **LAM11-ZWE.**
35	If presence of spacers 8, 15, 25, 31, absence of spacers 9-14, 21-24, 33-36, then **LAM12-Madrid1.**
36	If presence of spacers 25, 31, absence of spacers 3, 13, 21-24, 33-36, then **LAM2.**
37	If presence of spacers 25, 31, absence of spacers 9-11, 21-24, 33-36, then **LAM3.**
38	If presence of spacers 25, 31, absence of spacers 21-24, 33-36, 40, then **LAM4.**
39	If presence of spacers 25, 31, absence of spacers 13, 21-24, 33-36, then **LAM5.**
40	If presence of spacers 25, 28, 30, 31, 32, absence of spacers 21-24, 29, 33-36, then **LAM6.**
41	If presence of spacer 25, 28, 31, absence of spacers 20-24, 26, 27, 33-36, then **Turkey.**
42	If because of presence of spacers 25, 26, 28, 31, absence of spacers 21-24, 33-36, then **LAM8.**
43	If absence of 21 to 24, 33 to 36, presence of 25 and 31, then **LAM.**
44	If presence of spacers 1-43, then **Manu-ancestor.**
45	If presence of spacers 33, 35, absence of spacer 34, then **Manu1**.
46	If presence of spacers 32, 35, absence of spacers 33, 34, then **Manu2.**
47	If presence of spacer 33, 37, absence of spacers 34-36, then **Manu3.**
48	If presence of 37, 38, absence of spacers 1-36, absence of spacers 39-43, then **Microti.**
49	If absence of spacers 1-3, 8-22, 39-43, then **PINI.**
50	If presence of spacers 7, 23, absence of spacers 1-3, 8-22, 39-43, then **PINI1.**
51	If presence of spacer25, absence of spacers 1-24 and 39-43, then **PINI2.**
52	If presence of spacers 30, 36, absence the rest of spacers, then **Canetti.**

53	If presence of spacers 8, 11, 31. absence of spacers 9, 10, 33-36, then **S.**
54	If presence of spacer 31, absence of spacers 33-36, then **T.**
55	If presence of spacers 6, 19, 31, absence of spacers 7-18, 33-36, then **T1-RUS2.**
56	If presence of spacers 31, 39, 41, absence of spacers 33-36, 40, then **T2.**
57	If absence of spacers 33-36, 40, 43, and presence of spacers 41 – 42, then **T2-Uganda.**
58	If presence of spacer 12, 14, 31, absence of spacers 13, 33-36, then **T3.**
59	If presence of spacers 9, 20 31, absence of spacers 10-19, 33-36, then **T3-ETH.**
60	If presence of spacers 4, 9, 12, 14, 31, absence of spacers 5-8, 13, 33-36, then **T3-OSA**.
61	If presence of spacers 18, 20, 31, absence of spacers 19, 33-36, then **T4.**
62	If presence of spacers 18, 20, 22, 25, 31, 37, 40, absence of 19, 23, 24, 33-36, 38-39, then **T4-CEU1.**
63	If absence of spacer 23, 33-36, presence of spacers 22, 24, 31, then **T5.**
64	If presence of spacers 19, 21, 22, 24, 31, absence of spacers 20, 23, 33-36, then **T5-Madrid2.**
65	If presence of spacers 14, 31, absence of spacers 15-24, 33-36, then **T5-RUS1.**
66	If presence of spacers 17, 19, 31, absence of spacers 18, 33-36, then **X1.**
67	If presence of spacers 17, 19, 31, absence of spacers 18, 33-36, 39-42, then **X2.**
68	If presence of spacers 3, 17, 19, absence of spacers 4-12, 18, 33-36, then **X3.**
69	If presence of spacers 18, 42, absence of spacers 19-41, then **ZERO.**

Supplement 2: CDC Rules used in KBBN.

The following rules, based on presence and absence of spacers in a spoligotype, are used to determine the CDC sublineage. The notation sum(sp33-36) > 0 means that the sum of spacers 33 to 36 are greater than 0. This is equivalent to saying that at least one of the spacers from 33 to 36 is greater than 0.

**No.**	**Description**
1	If absence of spacers 3, 20, 21, 29-32, 34, sum(sp33-36) > 0, and presence of spacers 2, 4, 19, 22, then **Indo-Oceanic – Manila.**
2	If absence of spacers 2, 3, 29-32, 34, sum(sp33-36) > 0, and presence of spacers 1, 4, then **Indo-Oceanic – India.**
3	If absence of spacers 26, 27, 29-32, 34, sum(sp33-36) > 0, and presence of spacers 25, 28, then **Indo-Oceanic – Vietnam.**
4	If absence of spacers 2-13, 29-32, 34, sum(sp33-36) > 0, and presence of spacers 1, 14, then **Indo-Oceanic – Mexico.**
5	If sum(sp29-32) > 0 and absence of spacers 33-36, 18, and presence of spacers 17, 19, then **X.**
6	If sum(sp29-32) > 0, absence of 21 – 24, 33 - 36 and presence of spacers 20, 25, then **LAM.**
7	If sum(sp29-32) > 0, absence of spacers 22 – 31, 33 -36 and<br><br>(sp30=1, sp31=0, sp32=1), or (sp25=1, sp22-31=0, sp32=1), then **Haarlem.**
8	If sum(sp29-32) > 0 and absence of spacers 9, 10, 33 -36 and presence of spacers 8, 11, then **S.**
